# Comparative In-Depth Investigation of Benchmark Graphene Oxides in the Perspective of Their Integration into Industrial Production Processes

**DOI:** 10.3390/nano15130980

**Published:** 2025-06-24

**Authors:** Georgios N. Mathioudakis, Glykeria A. Visvini, Labrini Sygellou, Amaia Soto Beobide, George A. Voyiatzis

**Affiliations:** Foundation for Research and Technology-Hellas (FORTH), Institute of Chemical Engineering Sciences (ICE-HT), Stadiou Str., GR-26504 Rio-Patras, Greece; glukeriavisvini@hotmail.com (G.A.V.); sygellou@iceht.forth.gr (L.S.); asoto@iceht.forth.gr (A.S.B.)

**Keywords:** graphene oxide, characterization, physicochemical properties, standardization, thermal stability, industry

## Abstract

The incorporation of commercial graphene oxide (GO) into composites offers significant improvements in mechanical, thermal, and electrical properties, making it a promising material for industrial applications. This study presents a comprehensive characterization analysis of five commercial GOs, using advanced techniques to evaluate their structural, chemical, and especially their behavior when submitted to thermal treatment. The aim is to enable the use of GO in industrial processes of particular technological importance, where its thermal stability/integrity is required, such as in polymer composites, electronic and energy storage devices, among others. Raman spectroscopy and attenuated total reflectance–Fourier-transform infrared (ATR-FTIR) spectroscopy are employed to examine the structural defects and functional groups of GOs, while X-ray diffraction (XRD) provides insight into the crystallinity and interlayer spacing. Thermogravimetric analysis (TGA) assesses the thermal stability, and X-ray photoelectron spectroscopy (XPS) offers detailed information on the surface chemistry and relevant elemental composition of GOs. Additionally, the temperature-dependent behavior of GOs is explored through temperature-dependent XRD and IR measurements to investigate the thermal expansion and functional group stability. The study highlights the critical role of oxygen-containing groups—such as epoxides, hydroxyls, and carboxyls—while variations in the type and concentration of these functional groups across commercial GOs could influence the compatibility and performance of nanocomposites. This research attempts to fill to some extent the gap in understanding how the unique properties of different commercial GOs can be strategically applied to meet specific industrial performance requirements, such as barrier properties, transport efficiency, or mechanical strength, among others.

## 1. Introduction

Graphene oxide (GO) is a chemically modified graphene; it presents a single atomic layer and is, thereby, classified as a 2D material [1,2,3]. GO has emerged as a key material in the field of materials science due to its exceptional properties, such as high mechanical strength, electrical conductivity, and optical transparency [4,5,6]. These features position GO as an ideal candidate for numerous advanced applications in various industries, including optoelectronics [7], energy, environment [8,9], transparent conductive films (TCFs) [10], biotechnology [11,12] and membrane applications [12,13,14,15,16,17]. Another promising application of GO is its use in the production of composite films. These GO-incorporated composite films possess enhanced mechanical, thermal, electrical and breathable or even barrier properties, making them ideal candidates for use in advanced materials for industrial applications [17,18,19,20]. Extensive research has already been conducted on the synthesis, deposition, and characterization of GO-containing films to optimize their performance [21,22].

As a derivative of graphene, GO is characterized by the presence of oxygen-based functional groups, such as hydroxyl (−OH), alkoxy (C-O-C), carbonyl (C=O), carboxylic acid (−COOH), and other oxygen-based functional groups at the sp2 carbon basal plane [23], which makes it highly versatile and functional for different applications [11]. In particular, the C/O atomic ratio is typically close to 2, indicating the high concentration of oxygen [3].

The structural properties of GO, such as its sheet-like morphology and the degree of oxidation, play a critical role in determining its dispersion behavior in polymer matrices [24,25]. GO sheets, due to their large surface area and oxygenated groups, are highly hydrophilic and can form stable dispersions in water or organic solvents, but their aggregation tendency often limits their effectiveness in composite systems. The ability to control the dispersion of GO is key to producing composite films with uniform properties and enhanced mechanical performance. Additionally, the surface functionalization of GO can influence its interaction with polymer matrices, further enhancing the compatibility and properties of the final composite [26]. Understanding the behavior of GO at the microscopic and molecular levels allows manufacturers to tailor the material properties and achieve the performance characteristics required for specific applications.

Characterization of GO is essential to assess its suitability for different industrial applications. The process of synthesizing composite films requires not only selecting appropriate materials but also thoroughly understanding the properties of the components involved. As it pertains for GO, this means understanding its surface chemistry, dispersion behavior, structural characteristics, and the way it interacts with polymer matrices. These factors significantly affect the performance of composite films in their final application, whether in flexible electronics [27], sensors, conductive coatings [13], lightweight structural materials [28], or breathable or barrier film [17]. Therefore, the detailed correlation of different characterization techniques applied to commercial GOs with a decisive comparative analysis is critical to ensure its effectiveness in the development of advanced materials [29,30,31,32,33,34,35].

Various characterization techniques are applied to determine the key properties of GO, including size, morphology, surface area, chemical composition, and dispersion behavior. Techniques such as scanning electron microscopy (SEM), transmission electron microscopy (TEM), attenuated total reflectance–Fourier-transform infrared spectroscopy (ATR-FTIR), X-ray diffraction (XRD), Raman spectroscopy, X-ray photoelectron spectroscopy (XPS), and surface area analysis (BET) are commonly used to provide a comprehensive understanding of GO’s structure and functional groups. Each of these techniques contributes valuable information about the material’s physical and chemical attributes, which are crucial for optimizing its performance in composite films [29,30,31,32,33,34,35].

This study aims to provide a comprehensive characterization analysis of five commercial GOs in the context of their use in industrial production processes. By evaluating their physical, chemical, and structural properties through a series of advanced characterization techniques, this analysis will offer valuable insights into the behavior of GO-based materials, highlighting the factors that contribute to their performance and optimization. Ultimately, this study seeks to contribute to the ongoing research into the commercialization of GO-enhanced composite materials, paving the way for their widespread use in a variety of industrial applications.

## 2. Materials and Methods

Five different commercial GOs were procured from (a) ACS Material, Medford, MA, USA (GO, CAS: 240220, Single Layer-H), (b) Williamblythe, Accrington, UK (GO, CAS: JC0558, powder), (c) Nanografi, Çankaya, Turkey (GO, CAS: 7782-42-5, Single Layer powder), (d) Abalonyx, Oslo, Norway (GO, CAS: 16002, freeze-dried beads), and (e) Abalonyx (GO, CAS: 18002, dry powder < 100 mesh) and were characterized by micro-Raman, XRD, ATR, TGA, and XPS techniques. The characteristics of the GOs, as provided by the commercial suppliers, are presented in detail in Table 1.

## 3. Characterization Techniques

Raman spectroscopy was used as a non-destructive chemical analysis technique, which provides detailed information about the chemical structure of all commercial GO samples. Raman spectra were recorded on a T-64000 (Jobin Yvon-Horiba, Palaiseau, France) micro-Raman system equipped with a 2D-CCD Symphony II detector. A DPSS laser (Cobolt Fandango TMISO laser, SE-Solna, Sweden) provided the excitation wavelength (514.5 nm). Dispersion and detection of the Raman photons were done by an 1800 grooves/mm grating. The laser with a power of 1 mW was focused on the samples by a 50× (NA = 0.55) microscope objective with a spot diameter of ~1.5 μm; the spectral resolution was ~1 cm^−1^.

X-ray diffraction (XRD) was used to identify the structure of all commercial GO samples. XRD measurements were carried out by using a Bruker D8 Advance diffractometer (Bruker Optics GmbH, Karlsruhe, Germany) equipped with a Cu lamp (λCuKa = 1.54046 Å) at a scanning rate of 0.5°/min over a range of 5–50° (2θ). Temperature-dependent XRD measurements (RT-250 °C) were carried out with the same XRD equipped with the XRK900 reactor chamber (Anton Paar GmBH, Graz, Austria).

The ATR-FTIR spectra of GOs were recorded on an Alpha-II Diamond ATR Spectrometer of Bruker Optics GmbH, Ettlingen, Germany.

Thermogravimetric analysis (TGA) measurements of the samples were performed using a TA 55 model at a heating rate of 10 °C/min under inert (N_2_) conditions and a flow rate of 20 mL/min.

X-ray photoelectron spectroscopy (XPS) analysis was performed in an ultra-high-vacuum (UHV) system equipped with a SPECS Phoibos 100-1D-DLD hemispherical electron analyzer (Specs GmbH, Berlin, Germany) and a non-monochromatized dual-anode Mg/Al X-ray source. The photoelectron spectra were recorded using the MgKα with a 1253.6 eV photon energy non-monochromatized source (300 W) and an analyzer pass energy of 10 eV, giving a full width at half maximum (FWHM) of 0.85 eV for the Ag3d5/2 line. The acquisition and fitting were realized with the commercial software SpecsLab Prodigy (4.113.1) (Specs GmbH, Berlin, Germany).

## 4. Results

### 4.1. Characterization of Commercial GO Samples

**Figure 1:**
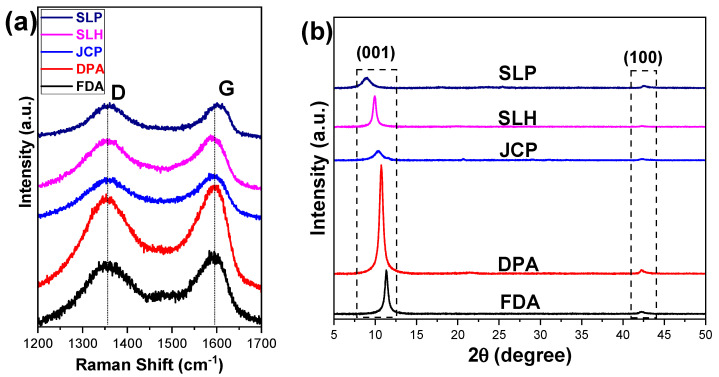
(**a**) Raman spectra of commercial GOs, (**b**) XRD diagrams of commercial GOs.

Raman spectroscopy is a powerful tool used to investigate the structure of graphene-based nanomaterials. Raman spectra of all commercial GO samples are shown in Figure 1. GO exhibits two strong characteristic bands at 1590 cm^−1^ (G band) and 1356 cm^−1^ (D band). The D peak corresponds to the breathing mode of the hexagonal rings, and its activation requires the presence of structural defects in the lattice. Therefore, the intensity of the D peak is often used as a measure of the degree of structural disorder. In addition, this specific peak has a direct dependence (its position) on the wavelength of the excitation line. The G band is attributed to the E2g phonon of the sp2 carbon bonding. The G peak corresponds to a doubly degenerated vibrational mode (of E2g symmetry) of stretch–compression of the C–C bonds of the hexagonal rings of the lattice. This peak is independent of the wavelength of the excitation monochromatic radiation and is particularly sensitive to the presence of strains and stresses in sp2 graphitic materials, due to the alteration of the symmetry of the hexagonal lattice [29,30,36,37,38,39].

The ratio of the intensities of the peaks D to G (I_D_/I_G_) is taken as an index of concentration of defects of the respective graphitic structure. The values obtained by processing the spectra of Figure 1 are listed in Table 2. From these values, it is observed that the GO FDA presents the fewest defects, while the GO SLP shows the highest concentration of defects in their lattice.

One of the most important features of graphene-based materials is the layer-to-layer distance in the graphene sheets. XRD is a very useful technique to determine the crystallinity of the graphene structure and to determine the layer-to-layer distance. The calculation of the changes in the interlayer d spacing value was carried out using Bragg’s Law Equation (1) [40]:

Bragg’s equation:

(1) 2d sin(θ) = nλ

where d is the distance between the crystallographic planes, θ is the XRD diffraction angle, and λ is the wavelength λCuKa = 1.54046 Å of the XRD Cu lamp.

Figure 1b shows the XRD diagrams of all commercial GOs. The main diffraction peak of GO is observed at angles from 2θ = 8.9–11.3° and corresponds to the crystallographic plane (001) of GO. The interlayer spacing (d-spacing) varies correspondingly to d~9.9–7.8 Å. The results indicate that the interlayer spacing was extended due to the intercalation of the oxygen-containing functional groups (hydroxyls (C-OH), carbonyls (C=O), epoxides (C-O), and carboxyls (O-C=O) into the layers, spacing the original crystals of the natural graphite samples. Also, a low-intensity diffraction peak appears at angles 42.1–42.5°, which corresponds to the crystallographic plane (100) of the hexagonal structure of graphite [38,39].

Table 2 summarizes the values of the interlayer spacing between the crystallographic planes, based on the Bragg Equation (1), of all GOs.

SLP and SLH GOs, although single layered, it is obvious that both display the characteristic peak (001) of GO at the smallest diffraction angles (2θ~8.9 and 9.9°, respectively), which corresponds to the largest distance between the crystallographic planes (d~9.9 and 8.9 Å, accordingly). In order to exhibit diffraction, both above GOs either (a) are easily exfoliable or/and (b) develop folds due to conformational changes that can occur in the single-layer graphene oxide [41], indicating a degree of order or stacking of the layers.

In the FDA GO, apparently the distances between the crystallographic planes are the smallest compared to all other GOs, which reveals that this specific GO contains the fewest oxygen-containing groups or those with the lowest affinity to water molecules.

Additionally, the full width at half maximum (FWHM) values for the 2θ peaks were calculated, as they provide information regarding well-ordered domains. The calculated FWHM values are as follows: SLP = 1.23, SLH = 0.45, JCP = 1.18, DPA = 0.53, and FDA = 0.46. Samples SLH, FDA, and DPA present narrower 2θ peaks, indicating a higher degree of structural order for these GO samples.

In a comparative comprehensive analysis of the results obtained from the Raman and XRD measurements, it is noticeable that more defects in the graphene oxide promote the extended interlayer distances. In other words, the less “defective” GO structure maintains a shorter interlayer distance. This can be further explained by correlating the degree of oxidation—reflected by functional groups—with the level of defects in each sample and how a higher density of functional groups, and thus more defects, may influence the interlayer distance.

**Figure 2:**
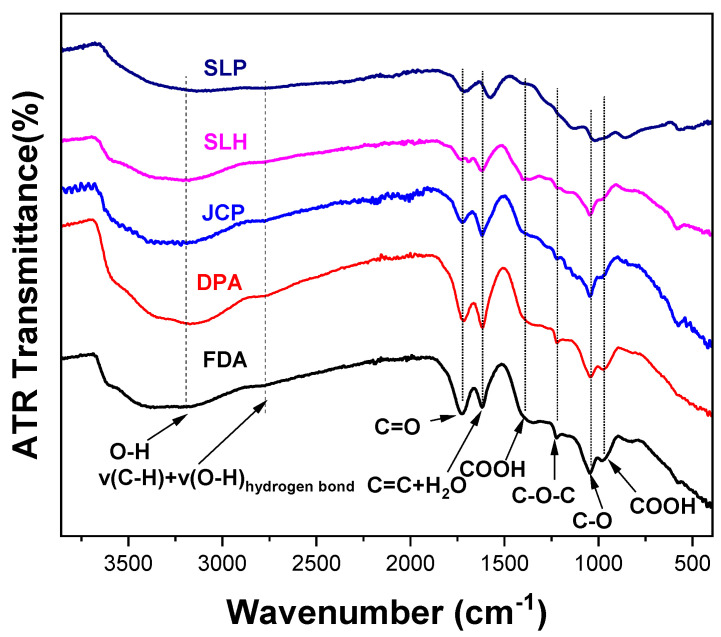
ATR-FTIR spectra of commercial Gos.

For the identification of the functional groups present in each sample, attenuated total reflection (ATR-FTIR) spectroscopy was performed. ATR-FTIR spectra were obtained in the range 4000–400 cm^−1^ (Figure 2). Peak assignments (Table 3) provided insights into the chemical composition of the samples and the presence or absence of oxygen-containing groups.

All GO samples show characteristic peaks associated with oxygen-containing functional groups at a higher or lower level. The strong broadened region at 3000–3500 cm^−1^ identifies the O-H modes, and the band at 1600 cm^−1^ is attributed to both C=C of the graphene layers and some contribution from adsorbed/intercalated H_2_O bending modes. Finally, the intense peak at 1720 cm^−1^ indicates the presence of carbonyl groups. Additional peaks at 1380, 1220, 1170, 1040, and 970 cm^−1^ are assigned to carboxyl, ether, and epoxide functional groups, as depicted in Table 3. The sample SLP, instead of a peak at ~1600 cm^−1^, it exhibits another one centered at 1580 cm^−1^; this corresponds to C=C bonds, indicating that there is no contribution from the 1616 cm^−1^ peak typically attributed to -OH groups. The minimal presence of -OH groups is further confirmed by a relatively weak band in the 3000–3500 cm^−1^ range. The FDA sample exhibits a higher concentration of C=O groups, as evidenced by the increased intensity of the peak at ~1700 cm^−1^ compared to the other GO samples. This is also accompanied by the highest intensity at 1380 cm^−1^, associated with carboxyl groups as well. All GO samples, as already stated, also contain other oxygen-containing groups. In particular, we can recognize that the SLH and JCP samples exhibit the strongest peak associated with epoxide groups (at 1040 cm^−1^).

The amount and the type of the oxygen-containing functional groups influence the interlayer spacing between the graphene sheets. This feature plays a significant role in the use of GO in a composite material with a specific application [31].

The TGA thermogravimetric profiles of the samples are also noteworthy, as they reveal the decomposition temperatures of various oxygen-containing groups. As such, TGA serves as a qualitative to semi-quantitative analytical tool, offering insights into the content of adsorbed water and oxygen-containing groups present on GO.

**Figure 3:**
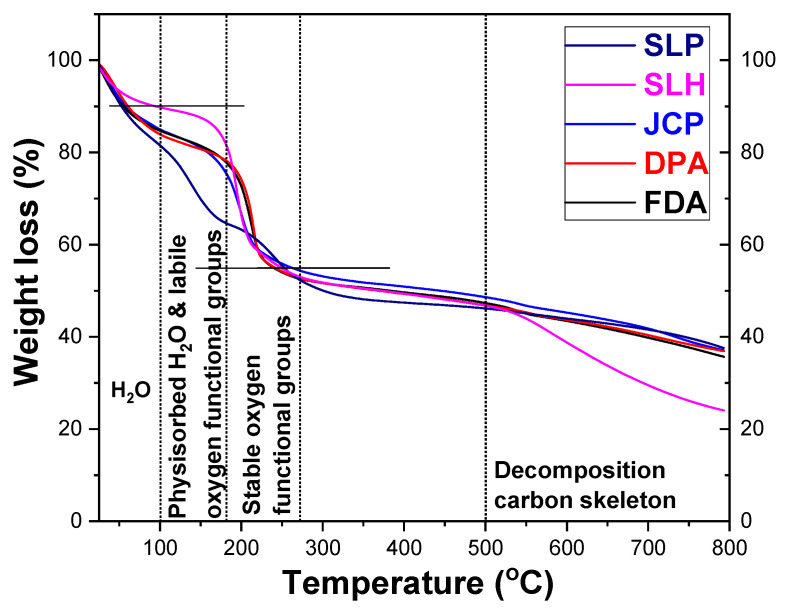
TGA thermographs of commercial GOs.

In the TGA thermographs presented in Figure 3, it is observed that GO is not a thermally stable material. The significant weight loss at a temperature below 100 °C is due to the loss of water molecules from the surface of GO, while at temperatures between 100 °C and 180 °C it is due to physiosorbed water and unstable oxygen-containing groups. The mass loss above 200 °C is due to the removal of the more resistant oxygen-containing groups [31,46,47,48]. Finally, the rapid mass loss starting at 500 °C can be attributed to the decomposition of the carbon skeleton of GO. From the TGA measurements (Figure 3), we can conclude that the weight loss observed at 270 °C for all GOs is approximately 40%, which likely originates from either adsorbed water or oxygen-containing functional groups. In other words, the functional groups present in all the GO samples account for approximately 40% of the total weight. SLP is the sample that exhibits the highest water loss, either from the surface of GO or from physisorbed water. It shows the lowest intensity of the IR band corresponding to C=O bonds (~1700 cm^−1^) and almost no detectable band associated with COOH groups (~1400 cm^−1^). Given that carboxyl groups retain water through both C=O and –OH interactions, it is rational to conclude that SLP, having a low concentration of these groups, has a reduced capacity to retain water. This is further supported by the fact that SLP is a single-layer material, where water is primarily absorbed on the surface, making it easier to lose compared to water trapped between layers in multilayer structures.

**Figure 4:**
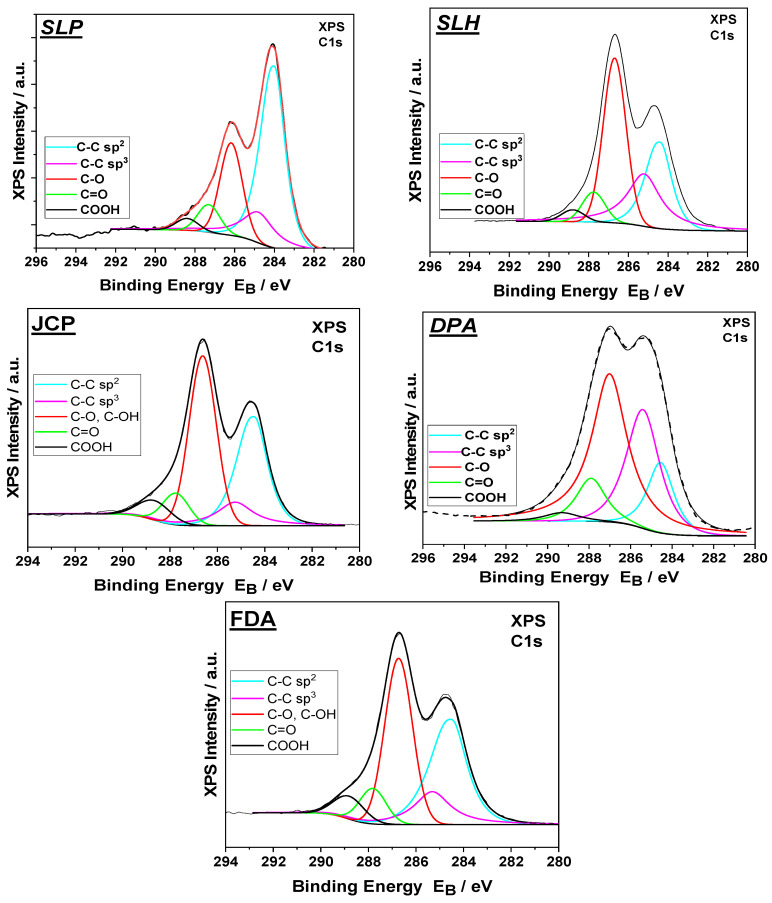
Deconvoluted C1s XPS spectra from all commercial GOs.

Along with ATR analysis, XPS spectra also provided complementary information related to the functional group composition (Figure 4). The deconvoluted C1s XPS spectra from all commercial GOs were analyzed into the following five components: C−C sp^2^ and defective sp^3^ bonds at binding energies (Table 4) of 284.3 ± 0.05 eV and 285.2 ± 0.05 eV, respectively; carbon−oxygen components at 286.6 eV assigned to both hydroxyls and epoxides (C−OH, C−O−C); carbonyls (C=O) at 288.1 eV; and carboxyls (COOH) at 289.1 eV [49]. The percentage component concentrations of the C1s and O1s peaks are also given in Table 4. From the peak areas of O1s, C1s, and S divided by the relative sensitivity factors and the energy analyzer transmission characteristics, the relative atomic concentrations are derived and are also presented.

The results of the XPS measurements are in agreement with the spectra obtained from the ATR-FTIR characterization, especially for the single-layer GOs, since there the surface information from the XPS can be better compared with the bulk information from the ATR. In the XPS, we observe that the GO SLP and SLH samples present a low % concentration of carbonyl and carboxyl groups. This is also obvious in the ATR-FTIR spectra by the low absorptions at the corresponding peaks at 970, 1380, and 1720 cm^−1^. The SLP sample exhibits a lower % concentration of hydroxyl and epoxide groups than the other single-layer sample, SLH. As we have already stated, SLP has almost no -OH group, as indicated by the absence of peak at 1616 cm^−1^ in the ATR spectrum. Once again, the low concentration of C–OH and C=O groups in SLP, as revealed by XPS measurements, accounts for the significant water loss observed in this sample during the TGA analysis. For the other samples, XPS measurements mostly confirm the type of oxygen-containing groups reported in the analysis of the ATR-FTIR spectra.

### 4.2. The Temperature Dependent Structural Differentiation in GO Morphology Using XRD and ATR-IR Measurements

The study of the temperature dependence of the structure of GOs was carried out via XRD and ATR-IR, aiming to determine any thermal-dependent structural variations. This information is particularly important because when incorporating GO into, e.g., a polymer matrix, it is useful to consider any thermally dependent structural variations.

**Figure 5:**
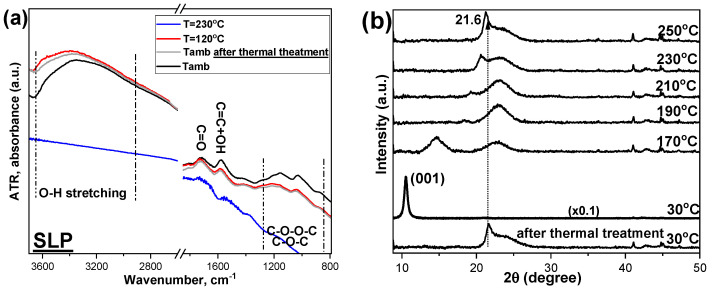

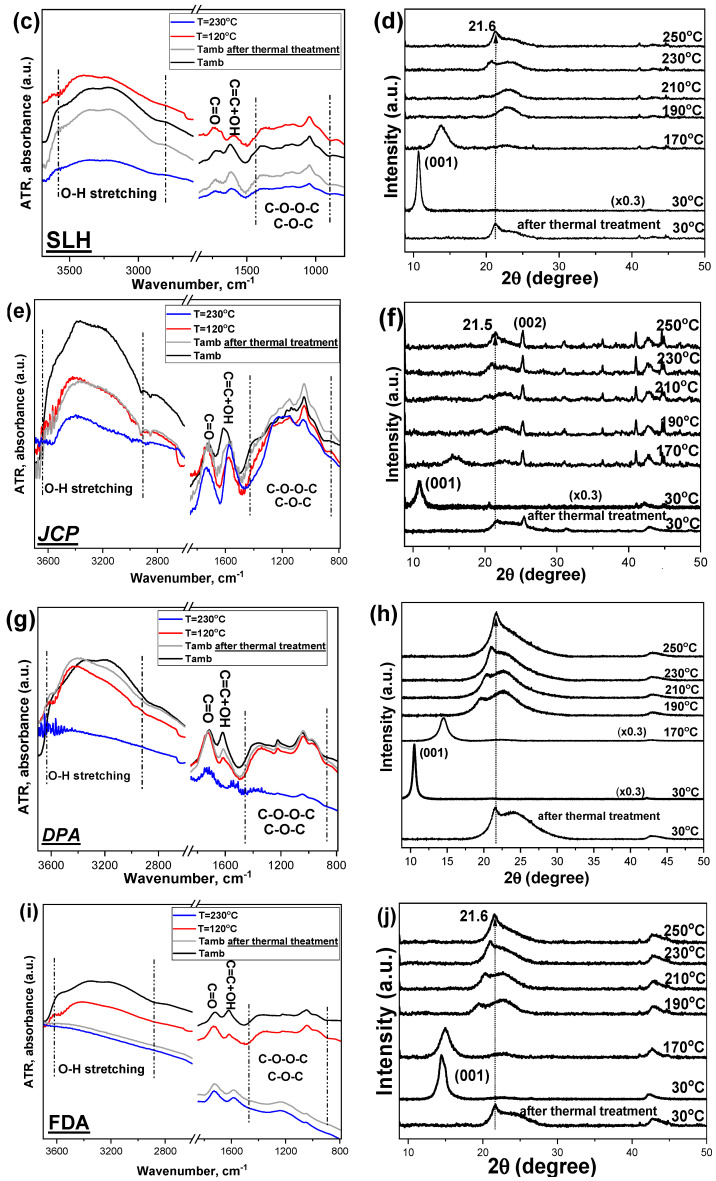
ATR-IR (**left column**) and XRD (**right column**) as a function of temperature of the commercial GOs: SLP (**a**,**b**), SLH (**c**,**d**), JCP (**e**,**f**), DPA (**g**,**h**), FDA (**i**,**j**). Some of the diffractograms have been multiplied by a factor (indicated on each graph) to clearly show the position of the peaks.

For the XRD study as a function of temperature (temperature range: RT to 250 °C), an XRK900 reactor chamber (Anton Paar GmBH) was integrated into the already existing XRD setup. In this context, XRD diagrams (Figure 5b,d,f,h,j) for each sample were received at six different temperatures (RT, 170, 190, 210, 230, and 250 °C), with subsequent cooling of the samples back to RT. In similar terms, ATR-IR study was monitored at three different temperatures (RT, 120 °C, and 230 °C). The samples were re-exposed to ambient conditions for 30 min, and then ATR spectra (Figure 5a,c,e,g,i) were again collected in order to clarify whether the GO samples subjected to this heat treatment are able to reabsorb some water molecules from the environment.

From the ATR-IR spectra of all samples (Figure 5, left column), it is observed that increasing the temperature to 120 °C leads to a progressive reduction in the intensity of specific vibration bands, such as the -OH stretching in the region ~3000–3500 cm^−1^. When the samples are heated up to 230 °C, the intensity of the -OH group bands is greatly reduced (JCP and SLH) or even vanishes (SLP, FDA, and DPA). During the heating of SLP, SLH, FDA and DPA, a decrease in the intensity of the bands corresponding to ethers, peroxides, epoxides, and ketones (~ 1400–800 cm^−1^) is observed. In the case of FDA and SLH the decrease in these functional groups is not so evident. As stated above, the band at ~1600 cm^−1^ is attributed to C=C bending vibrations of the graphene layers (1580 cm^−1^) and to adsorbed/interfered water molecules (1616 cm^−1^). For all samples, it is obvious the evaporation of naturally adsorbed water at a temperature of 120 °C (red spectra), while the band corresponding to carbonyl (C=O) at ~1720 °C remains essentially unaffected. However, when samples are heated to 230 °C (blue spectra), a decrease in carbonyls (C=O) ~1720 cm^−1^ and carboxyls (COOH) ~1380 cm^−1^ band intensity is noticed, with the FDA and JCP samples being the least affected. For the other samples, the carbonyls (C=O) ~1720 cm^−1^ and carboxyls (COOH) ~1380 cm^−1^ are not particularly temperature-dependent. Finally, when all the GO samples are re-exposed at room temperature conditions (grey spectra), SLP, SLH, and JCP samples recover to some extent the intensity for the 3000–3500 cm^−1^ band achieved for samples prior to thermal treatment, and also a slight increase in the intensity of the peaks associated with –OH groups at 1600 cm^−1^ is also observed, indicating the presence of functional groups capable of adsorbing water. This behavior is consistent across all samples except for FDA.

Regarding XRD measurements as a function of temperature (Figure 5, right column), all GOs at RT conditions present the main diffraction peak at angles in the range 2θ = 8.9–11.3° and correspond to the crystallographic plane (001). The exact value of this angle defines the distance between the crystallographic planes, as established in the previous section. This is mainly attributed to the adsorption of water due to the presence of hydrophilic functional oxygen-containing groups. After the heat treatment at 170 °C, the intensity of the specific peak shifts, decreases, and becomes broader due to the GO reduction starting to some extent. With further increasing temperature, this peak shifts to 2θ = 19.5–21.6°, which corresponds to a crystallographic lattice spacing of 4.5 to 4.1 Å for temperatures of 190 °C to 250 °C and is accompanied by a broad shoulder. As such, the reduction of GO is effectively demonstrated. The peak observed for reduced GO is attributed to the interlayer spacing between adjacent graphene layers. The GO samples that underwent heat treatment up to 250°C, when allowed to return to ambient temperature (30°C), did not show significant changes in their XRD pattern.

Τhe T-dependent ATR spectra indicate that with increasing temperature to 120 °C and 230 °C, the primary change is the loss of water molecules, while for some GO samples, a partial decomposition of oxygen-containing groups is also initiated. Furthermore, the XRD and TGA results reveal that from 170 °C onward, a progressive loss of oxygen-containing groups occurs with increasing temperature, leading ultimately, at 250 °C, to the transformation of the initial GO into reduced graphene oxide (rGO) and a graphite-like structure.

Table 5 aims to summarize all the results obtained from the techniques used to characterize the five commercial GOs.

In summary, among the multilayer GO samples, JCP appears to a have high content of carbonyl and carboxyl groups and the largest interlayer spacing, being stable to temperature treatment up to 200 °C, while FDA shows the smallest interlayer distance and high concentration of carboxyl groups. In GO, carboxyl groups are mainly located at the edges of the nanosheets, whereas hydroxyl and epoxy groups are typically distributed across the basal plane, which constitutes the main surface [50,51]. A relevant application of the benchmarked GOs could be to the technical fabrics for the construction of the roofing membranes, especially for pitched roofs, to avoid the accumulation of moisture and condensation. The water vapor permeable (WVP) membranes and the vapor control layers (VCL), based on polyolefin, may benefit from the incorporation of the GO, since, depending on the weight fraction of GO, they could present higher or lower breathability potential, substituting, e.g., the benchmark stretched CaCO_3_ (calcium carbonate) micro/nano particles containing films. From the results of the temperature-dependent experiments (Figure 5), the ATR spectra indicate that there are groups, such as carbonyls, that withstand heat treatment, while in the XRD spectra, graphene oxide undergoes a degree of dehydration with a shift of the main diffraction peak to higher angles without converting into reduced graphene oxide at least up to 210 °C [43], perhaps up to 230 °C. In the specific application, our choice would be the GOs that are not single layered; that is, the JPC, FDA, and DPA, with a preference for the FDA that sustains its initial integrity till 170 °C. In general, an increased concentration of carboxyl groups enhances water dispersibility, facilitates chemical affinity to biomolecules, and increases surface reactivity for specific uses. Carboxyl groups also contribute to GO’s hydrophilicity and enable interactions with other molecules via hydrogen bonding and electrostatic forces. On the other hand, hydroxyl groups improve interactions like adsorption and cross-linking through hydrogen bonding, impacting material compatibility and performance. Additionally, epoxy groups, especially on the basal plane, are useful in cross-linking reactions, making GO suitable for composite materials, particularly when combined with epoxy resins. By carefully controlling the synthesis and functionalization of GO, its properties can be tailored to meet the requirements of specific applications. Ultimately, the selection of an optimal graphene oxide depends on the specific requirements of the nanocomposite application, and therefore the insights of the in-depth characterization of the different GOs are essential for optimizing the composite.

## 5. Conclusions

This comprehensive analysis of five commercial graphene oxide (GO) samples has provided valuable insights into their structural, chemical, and thermal characteristics, which are critical for their successful integration into industrial processes. The combined use of Raman, ATR-FTIR, XRD, TGA, and XPS techniques enabled a detailed evaluation of the defect density, functional group distribution, crystallinity, thermal stability, and surface chemistry of each GO. Furthermore, temperature-dependent XRD and IR analyses revealed important information about thermal expansion behavior and the stability of oxygen-containing functional groups under thermal stress. The findings underscore the significant variability among commercial GOs and highlight how these variations directly impact their compatibility and performance in composite applications. By precisely controlling the synthesis and functionalization of GO, its properties can be tailored to suit the specific needs of various applications. This study highlights the necessity of a similar thorough characterization of the materials before their use/integration into industrial processes to select the most suitable materials or their mixture for optimization.

## Figures and Tables

**Table 1:** List of commercial GO samples.

Code	Commercial GOs	Company	Particle Diameter	Thickness
SLH	Single Layer-H	ACS Material	1–5 μm	0.8–1.2 nm
JCP	JC05 58 powder	Williamblythe	variable	
SLP	Single Layer powder	Nanografi	2 μm	1 nm
FDA	Freeze dried beads	Abalonyx	4–5 mm	
DPA	Dry Powder < 100 mesh	Abalonyx		

**Table 2:** Information extracted from the Raman spectra and the XRD diagrams of all GOs.

Commercial GOs	Raman	XRD	
	${I=I}_{D}/I_{G}$	2θ (°)	d (Å)
SLP	0.97 ± 0.02	8.9	9.9
SLH	0.95 ± 0.03	9.9	8.9
JCP	0.91 ± 0.02	10.4	8.5
DPA	0.89 ± 0.02	10.8	8.2
FDA	0.86 ± 0.04	11.3	7.8

**Table 3:** IR peak assigment of commercial GOs [32,42,43,44,45].

Wavenumber (cm^−1^)	Assignment	Oxygen-Containing Groups
3000–3500 (broad)	O-H	hydroxyls
2773	v(C-H) + v(O-H) hydrogen bond	
1720	C=O	carbonyls
~1600	H_2_O (1616 cm^−1^)	water
	C=C (1580 cm^−1^)	“graphene layers”
1380	C-OOH	carboxyls
1220	C-O-C	ethers
~1170	C-O	epoxides
1040	C-O	epoxides
970	COOH	carboxyls

**Table 4:** The percentage of component concentration of C1s derived from XPS C1s peak deconvolution and % relative atomic concentration.

	C1s Components					Atomic Concentration		
	C-C sp^2^	C-C sp^3^	C-O/ C-OH	C=O Carbonyl	COOH Carboxyl	O	C	S
Binding Energy (eV)	284.4	285.2	286.7	287.8	288.8	532.4	-	168.3
SLP (% Conc.) *	52.5	15.8	22.2	6.7	2.8	34.0	56.5	5.8
SLH (% Conc.)	25.3	26.7	38.3	7.0	2.7	33.1	65.5	1.4
JCP (% Conc.)	33.3	11.2	42.2	7.6	5.8	32.7	66.0	1.3
DPA (% Conc.)	13.1	27.6	48.4	8.9	2.0	30.1	69.9	-
FDA (% Conc.)	32.4	16.9	36.6	8.5	5.5	33.2	69.8	1.0

* Fluorine 2.5% and calcium 1.2% are also detected.

**Table 5:** Summary of commercial GO samples characterization.

Benchmark GOs	Raman and XRD	TGA	XPS	T-Dependent ATR	T-Dependent XRD
SLP	Higher defects and interlayer distance	Higher H_2_O physiosorbed	Lowest C=O content	Low -OH content	
SLH		Lower H_2_O physiosorbed	Lowest COOH content		
JPC			Highest COOH content	Quite stable at high T	Workable till ~200 °C
DPA			Highest C=O content	Low -OH content	Workable till ~200 °C
FDA	Lower defects and interlayer distance			High C=O content	Stable till 170 °C and workable till ~200 °C

A note is made of the fact that XPS mainly measures the oxygen containing groups located on the GOs surface.

## Data Availability

The raw data will be available from corresponding author upon reasonable request.
